# Association Between ACL Continuity on Magnetic Resonance Imaging at 5 Years After an Acute ACL Rupture and 11-Year Outcomes: A Secondary Analysis From the KANON Trial

**DOI:** 10.1177/03635465251339061

**Published:** 2025-05-19

**Authors:** Stephanie R. Filbay, Frank Roemer, Ewa M. Roos, Aleksandra Turkiewicz, Richard Frobell, L. Stefan Lohmander, Martin Englund

**Affiliations:** † Centre for Health, Exercise and Sports Medicine, Department of Physiotherapy, University of Melbourne, Melbourne, Victoria, Australia; ‡ Department of Radiology, Friedrich-Alexander University of Erlangen-Nuremberg, Erlangen, Germany; § Department of Radiology, Boston University School of Medicine, Boston, Massachusetts, USA; ‖ Department of Sports Science and Clinical Biomechanics, University of Southern Denmark, Odense, Denmark; ¶ Clinical Epidemiology Unit, Department of Clinical Sciences, Lund University, Lund, Sweden; # Orthopaedics Division, Department of Clinical Sciences, Lund University, Lund, Sweden; Investigation performed at Lund University, Lund, Sweden

**Keywords:** anterior cruciate ligament reconstruction, rehabilitation, osteoarthritis, magnetic resonance imaging

## Abstract

**Background:**

Emerging evidence suggests that anterior cruciate ligament (ACL) ruptures can restore ACL fiber continuity. The relationship between ACL continuity on magnetic resonance imaging (MRI) (sign of ACL healing) and outcomes >5 years after an acute ACL rupture has not been investigated.

**Purpose:**

This study aimed to (1) describe clinical outcomes and radiographic osteoarthritis (ROA) at 11 years based on ACL continuity status at 5 years and (2) investigate the relationship between 5-year ACL continuity status and 11-year Knee Injury and Osteoarthritis Outcome Score (KOOS_4_) scores.

**Study Design:**

Secondary analysis of KANON randomized controlled trial; Level of evidence, 3.

**Methods:**

Overall, 105 of 121 (87%) active adults with acute ACL ruptures randomized to undergo initial exercise therapy and optional delayed ACL reconstruction (ACLR) or early ACLR and postoperative exercise therapy completed 11-year follow-up. MRI scans at 5 years were evaluated using the Anterior Cruciate Ligament OsteoArthritis Score (0-3), with grades 0 to 2 considered to represent “ACL continuity.” Patient-reported outcomes (KOOS_4_, 36-Item Short Form Health Survey, Tegner Activity Scale, self-reported new knee injuries), knee laxity, and radiographic findings (tibiofemoral and/or patellofemoral ROA) were assessed at 11 years. The relationship between 5-year ACL continuity and 11-year KOOS_4_ scores (0-100) was examined using linear regression, adjusted for age, sex, smoking, and baseline KOOS_4_ scores.

**Results:**

Of patients managed nonsurgically, 58% (n = 14) had ACL continuity and 42% (n = 10) had ACL discontinuity at 5 years. Analyses suggest that ACL continuity was associated with worse 11-year KOOS_4_ scores compared with delayed ACLR (adjusted mean difference, –20.2 [95% CI, –31.9 to −8.6]) and early ACLR (adjusted mean difference, –15.5 [95% CI, –26.4 to −4.7]) as well as similar or worse KOOS_4_ scores compared with ACL discontinuity (adjusted mean difference, –11.4 [95% CI, –26.5 to 3.6]). The proportion of patients with tibiofemoral ROA ranged from 14% (ACL continuity) to 23% (delayed ACLR), and the proportion of patients with patellofemoral ROA ranged from 11% (ACL discontinuity) to 41% (early ACLR).

**Conclusion:**

ACL continuity on 5-year MRI may be associated with worse patient-reported outcomes at 11 years after an ACL injury compared with early or delayed ACLR.

**Registration:**

84752559 (ISRCTN)

The ability of a ruptured anterior cruciate ligament (ACL) to heal without surgical management is an emerging area of research receiving attention among researchers, clinicians, and the general public.^5,6^ A recent secondary analysis of KANON trial data suggested that 30% of participants with acute ACL ruptures who were randomized to management with initial exercise therapy had ACL continuity on magnetic resonance imaging (MRI) at 2 years after the injury.^6^ This finding challenges the common assumption that a ruptured ACL has limited or no healing capacity. Additionally, patients with ACL continuity on 2-year MRI reported better 2-year patient-reported outcomes than those with ACL discontinuity and those managed with early or delayed ACL reconstruction (ACLR).^6^ This finding gives rise to the possibility that the healing status of the ACL could help to explain why some patients do well when managed with rehabilitation alone (historically labeled as “copers”) while others do not achieve satisfactory knee function and decide to undergo delayed ACLR (historically labeled as “noncopers”). The coper/noncoper terminology used to describe successful and unsuccessful outcomes when managed with rehabilitation alone implies that patients can cope with an “ACL-deficient” knee by achieving knee stability through neuromuscular control and strengthening (reflecting the historical view that ACL ruptures do not heal).^12,17^ Therefore, studies aiming to differentiate copers from noncopers to inform treatment decision-making have not considered the healing status of the ACL as a potential determinant of outcomes.^12,17^ More evidence is needed to better understand the relationship between signs of ACL healing observed on MRI (eg, ACL continuity or discontinuity) and patient outcomes including knee function, symptoms, quality of life, and radiographic osteoarthritis (ROA).

Long-term 11-year follow-up data from the KANON trial are now available.^14^ In line with 2- and 5-year findings, there was no important difference in patient-reported outcomes at 11 years between treatment groups (early ACLR vs initial exercise therapy with optional delayed ACLR).^14^ It is possible that regaining continuity of the native ACL after nonsurgical treatment is associated with fewer signs of knee osteoarthritis, better knee function, and fewer symptoms more than 10 years after the injury. At 11-year follow-up, 44% of KANON trial participants had developed ROA in their ACL-injured knee in the tibiofemoral and/or patellofemoral joint.^14^ Investigating continuity of the ACL in relation to 11-year outcomes may provide new insights into the clinical relevance of signs of regained ACL continuity as observed on MRI. The present study aimed to (1) describe clinical outcomes and ROA at 11 years based on ACL continuity status at 5 years and (2) investigate the relationship between 5-year ACL continuity status and 11-year Knee Injury and Osteoarthritis Outcome Score (KOOS_4_) scores.

## Methods

### Study Design

This exploratory analysis used data from the KANON trial (ISRCTN 84752559), a randomized controlled trial in which 121 adults with acute ACL ruptures were randomized to management with initial exercise therapy and optional delayed ACLR or early ACLR and postoperative exercise therapy.^7,8^ The study was approved by the ethics committee of Lund University (LU 535-01).

### Participants

The 121 participants with acute ACL ruptures who were included in the KANON trial were aged 18 to 35 years and engaged in moderate activities or nonprofessional sports (scores 5-9 on Tegner Activity Scale) at the time of the ACL rupture.^7,8^ An acute ACL rupture was confirmed by MRI for all but 1 participant (who had a positive pivot-shift test finding), and randomization occurred within 4 weeks of the ACL rupture.^7,8^ Patients were ineligible for the trial if they had a full-thickness cartilage lesion, had undergone extensive meniscal fixation (fixation of meniscocapsular separation ≥10 mm, requiring a change in the postoperative rehabilitation protocol), or had total collateral ligament ruptures.^7,8^ All ACLR procedures were performed using a single-bundle hamstring tendon or patellar tendon autograft, in line with the preference of 4 senior surgeons who carried out all procedures.

### Criteria for ACL Continuity on MRI

Knee MRI was performed at 5 years using a 1.5-T system (Gyroscan Intera; Philips) with a circular polarized surface coil using identical sequences for all participants. MRI scans were evaluated using the Anterior Cruciate Ligament OsteoArthritis Score (ACLOAS) to assess ACL continuity (considered a surrogate for ACL healing) by an experienced musculoskeletal radiologist (F.R.). The ACLOAS contains criteria for grading continuity of the ACL, with grades from 0 to 3^15^:

- 0 = healthy ligament with hypointense signal and regular thickness and continuity,
- 1 = thickened ligament and/or high intraligamentous signal with normal course and continuity,
- 2 = thinned or elongated but continuous ligament, and
- 3 = absent ligament or complete discontinuity.

For the primary analysis, ACLOAS grades were dichotomized to 0-2 (ACL continuity on MRI) and 3 (ACL discontinuity on MRI).

### Follow-up Assessments

At 11 years, participants completed follow-up assessments including patient-reported outcome measures (KOOS_4_, Tegner Activity Scale, self-reported new injury to index knee between 5 and 11 years), knee laxity testing, and knee radiography.^14^ A comparison of 11-year outcomes between the 2 randomized treatment groups (ie, initial exercise therapy with optional delayed ACLR vs early ACLR with postoperative exercise therapy) was published in 2023.^14^ The 2- and 5-year outcomes in relation to ACL continuity status have been reported in a previous study.^6^

#### Knee Injury and Osteoarthritis Outcome Score

The KOOS_4_, an aggregate measure of the 4 most relevant KOOS subscales in patients with ACL injuries, was used as the primary outcome for the KANON trial. It provides a score ranging from 0 (worst) to 100 (best) based on responses to the pain, symptoms, sports and recreation, and quality of life subscales. The KOOS_4_ is recommended as a primary outcome measure for use in clinical trials because it examines 4 core outcome domains (pain, symptoms, sports and recreation, and quality of life).^20^

#### Radiographic Osteoarthritis

Radiographs were assessed at 11-year follow-up according to the Osteoarthritis Research Society International atlas, by an experienced radiologist blinded to group allocation.^1^ ROA of the tibiofemoral or patellofemoral joint was defined as ≥1 compartment with grade ≥2 joint space narrowing (tibiofemoral only), sum of marginal osteophytes grade ≥2 in the same compartment, and/or grade 1 joint space narrowing in combination with grade 1 osteophytes in the same compartment (tibiofemoral only). A summed score of radiographic features (grades of joint space narrowing and grades of osteophytes) in the index knee (tibiofemoral and patellofemoral joints) was also calculated for each participant, with possible scores ranging from 0 (no radiographic signs of osteoarthritis) to 30 (severe radiographic signs of osteoarthritis).^14^

#### KOOS Subscales

Other measures included individual KOOS subscales (assessed at baseline and 2, 5, and 11 years) and the proportion of participants meeting the Patient Acceptable Symptomatic State (PASS) and treatment failure cutoff for each KOOS subscale at 11-year follow-up. These cutoffs were calculated in a random sample from the Norwegian Knee Ligament Register at 6 to 24 months after ACLR.^10^ The question used to assess the PASS was as follows: “Considering your knee function, do you feel that your current state is satisfactory? With knee function, you should take into account all activities during your daily life, sport and recreational activities, your level of pain and other symptoms, and also your knee-related quality of life.”^10^ The following question was used to assess treatment failure: “If you answered ‘No’ to the previous question, would you consider your current state as being so unsatisfactory that you think the treatment has failed?”^10^

#### 36-Item Short Form Health Survey (SF-36)

The SF-36 was used to capture valid and reliable information about functional health and well-being.^19^ The SF-36 measures 8 health domains, enabling the calculation of 2 summary scores: the physical component summary (physical functioning, bodily pain, general health perceptions, and physical role limitation) and the mental component summary (vitality, emotional role functioning, social role functioning, and mental health).^19^

#### Tegner Activity Scale

Activity level was assessed with the Tegner Activity Scale^16^ completed at baseline and at 2-, 5-, and 11-year follow-up. Scores range from 0 (disability pension because of knee) to 10 (competition in elite sport). The Tegner Activity Scale has good test-retest reliability (0.82-0.92) when used in patients with a knee injury.^3^

#### Knee Laxity

Knee laxity was assessed at 11 years using the Lachman test (grade 0 or 1 = “normal” or “nearly normal”)^13^ and the pivot-shift test (grade 0 or 1 = “normal” or “nearly normal”).^11,18^ The method of assessments has been reported.^7^

#### New Knee Injuries

New knee injuries sustained between 5- and 11-year follow-up were self-reported by participants at 11 years. This method of assessment has limitations and is subject to recall bias. Although participants reporting new knee injuries were asked to describe them in detail using an open-text response, the type and severity of injury were not always clear. Therefore, in this article, we report only those injuries that resulted in a consultation with a health care professional (ie, primary care clinician, physical therapist, and/or orthopaedic surgeon), in line with the publication of 11-year outcomes.^14^

### Statistical Analysis

Participants were assigned to 1 of 4 groups based on 5-year ACL continuity and treatment status (managed with exercise therapy alone with ACL continuity, managed with exercise therapy alone with ACL discontinuity, commenced initial exercise therapy but underwent delayed ACLR before 11 years, and underwent early ACLR and postoperative exercise therapy within 10 weeks of the injury), consistent with our earlier study.^6^ The 11-year outcomes were reported descriptively as the mean ± standard deviation or median (interquartile range) as appropriate. PASS and treatment failure thresholds were applied to 11-year KOOS subscale scores to assist with the interpretation of findings. The relationship between 5-year ACL continuity status and 11-year KOOS_4_ scores was examined using linear regression, adjusted for age, sex, smoking, and baseline KOOS_4_ scores. The model fit was assessed, and to account for a ceiling effect in KOOS_4_ scores, a sensitivity analysis was performed using a censored regression (tobit) model.

There were 4 participants who were missing 5-year MRI data. A previous study found that ACL continuity status remained constant between 2- and 5-year follow-up.^6^ For this reason, 2-year ACL continuity status was used to estimate the 5-year ACL continuity status for these 4 participants (2 had ACL continuity, and 2 had ACL discontinuity, at 2 years). One participant had missing 2- and 5-year MRI data and was excluded from this study.

## Results

Data from 105 participants (87%) were available for analysis. Of those managed nonsurgically, 58% (n = 14) had ACL continuity and 42% (n = 10) had ACL discontinuity at 5 years. Participant characteristics are reported in Table 1.

**Table 1:** Participant Characteristics*^*a*^*

		Exercise Therapy Alone			
	All (n = 105)	ACL Continuity (n = 14)	ACL Discontinuity (n = 10)	Exercise Therapy + Delayed ACLR (n = 29)	Early ACLR + Exercise Therapy (n = 52)
Age at 11 y, mean ± SD, y	37 ± 5	38 ± 5	37 ± 5	36 ± 5	37 ± 5
Female sex	30 (29)	5 (36)	2 (20)	11 (38)	12 (23)
Weight at baseline, mean ± SD, kg	75 ± 13	74 ± 16	78 ± 13	72 ± 9	77 ± 14
Weight at 11 y, mean ± SD, kg	79 ± 15	78 ± 18	80 ± 14	76 ± 12	81 ± 16
Height at baseline, mean ± SD, cm	177 ± 8	174 ± 9	180 ± 7	175 ± 7	177 ± 8
Current or former smoker	31 (30)	2 (14)	5 (50)	8 (28)	16 (31)
History of contralateral ACL injuries	12 (11)	2 (14)	00 (0)	2 (7)	8 (15)
Concomitant meniscal injury	58 (55)	9 (64)	8 (80)	11 (38)	30 (58)
Meniscal surgery before 5 y	51 (49)	6 (43)	8 (80)	16 (55)	22 (42)
KOOS_4_ score at baseline					
Mean ± SD	36 ± 14	36 ± 14	36 ± 13	35 ± 11	37 ± 16
Median (IQR)	34 (27-41)	35 (26-44)	39 (28-43)	32 (26-39)	34 (27-41)
Preinjury Tegner activity score					
Mean ± SD	8 ± 1	8 ± 1	8 ± 1	8 ± 1	8 ± 1
Median (IQR)	9 (7-9)	8 (7-9)	9 (7-9)	9 (7-9)	9 (7-9)
No. of rehabilitation visits					
Mean ± SD	60 ± 36	32 ± 9	48 ± 34	67 ± 37	67 ± 37
Median (IQR)	59 (35-78)	34 (27-39)	37 (17-73)	64 (36-84)	58 (43-84)
Anterior tibial stump dislocation on MRI	N/A	1 (7)	00 (0)	8 (28)	N/A

a Data are shown as n (%) unless otherwise indicated. Baseline MRI scans were not assessed in the early ACLR group. ACL, anterior cruciate ligament; ACLR, anterior cruciate ligament reconstruction; IQR, interquartile range; KOOS, Knee Injury and Osteoarthritis Outcome Score; MRI, magnetic resonance imaging; N/A, not applicable.

**Figure 1.**
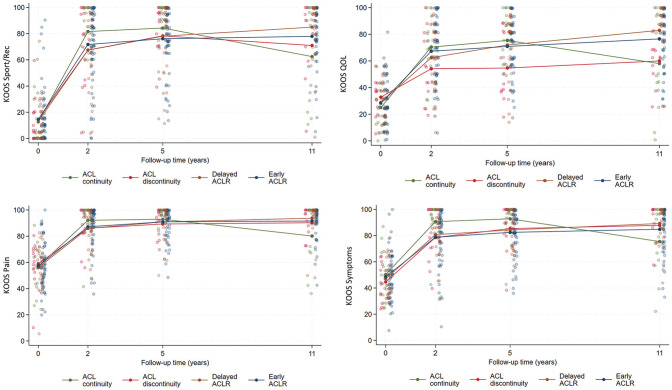
Individual and group mean values of Knee Injury and Osteoarthritis Outcome Score (KOOS) subscale scores at baseline and 2, 5, and 11 years’ follow-up by 5-year anterior cruciate ligament (ACL) continuity and treatment status. ACLR, anterior cruciate ligament reconstruction; QOL, quality of life; Sport/Rec, sports and recreation.

**Figure 2.**
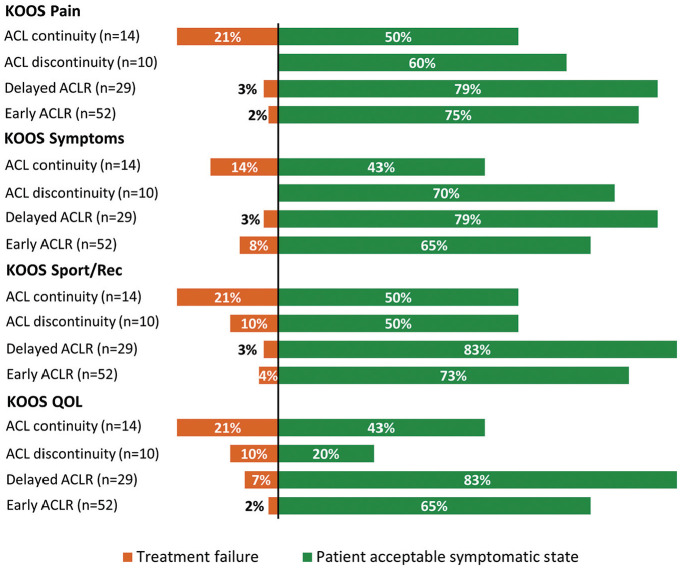
Proportion of participants meeting the Patient Acceptable Symptomatic State (PASS) and treatment failure thresholds for Knee Injury and Osteoarthritis Outcome Score (KOOS) subscales at 11 years. Thresholds (PASS and treatment failure, respectively): KOOS pain (89 and 57), KOOS symptoms (83 and 56), KOOS sports and recreation (Sport/Rec) (72 and 57), and KOOS quality of life (QOL) (73 and 28). ACL, anterior cruciate ligament; ACLR, anterior cruciate ligament reconstruction.

The 11-year outcomes are presented in Table 2, and Figure 1 depicts the change in group mean values of KOOS subscale scores over time (baseline and 2, 5, and 11 years’ follow-up). Also, 43% to 50% of patients with ACL continuity at 5 years met the PASS for the different KOOS subscales at 11 years, and 14% to 21% met the criteria for treatment failure (Figure 2). In comparison, 65% to 83% of patients managed with early or delayed ACLR met the PASS, and 2% to 8% met the criteria for treatment failure for a given KOOS subscale.

**Table 2:** 11-Year Outcomes*^*a*^*

	Exercise Therapy Alone		Exercise Therapy + Delayed ACLR (n = 29)	Early ACLR + Exercise Therapy (n = 52)
	ACL Continuity (n = 14)	ACL Discontinuity (n = 10)		
KOOS_4_, mean ± SD	69 ± 29	77 ± 17	88 ± 16	83 ± 17
Sports and recreation				
Mean ± SD	63 ± 36	71 ± 31	85 ± 22	78 ± 22
Median (IQR)	70 (30-100)	80 (55-95)	95 (80-100)	80 (68-95)
Quality of life				
Mean ± SD	58 ± 35	60 ± 18	83 ± 20	77 ± 22
Median (IQR)	50 (38-94)	66 (50-69)	88 (75-94)	81 (62-94)
Pain				
Mean ± SD	80 ± 24	90 ± 11	94 ± 12	92 ± 12
Median (IQR)	93 (67-100)	94 (83-100)	100 (94-100)	97 (90-100)
Symptoms				
Mean ± SD	76 ± 25	88 ± 15	89 ± 14	85 ± 17
Median (IQR)	77 (61-96)	93 (75-100)	96 (86-100)	93 (75-98)
SF-36				
Physical component summary				
Mean ± SD	74 ± 29	83 ± 16	91 ± 12	87 ± 14
Median (IQR)	82 (42-98)	85 (79-94)	97 (84-99)	90 (80-98)
Mental component summary				
Mean ± SD	83 ± 18	80 ± 20	90 ± 10	89 ± 8
Median (IQR)	86 (81-93)	92 (68-95)	93 (89-96)	90 (87-95)
Tegner				
Mean ± SD	3 ± 2	4 ± 3	5 ± 2	5 ± 2
Median (IQR)	4 (2-4)	4 (2-4)	5 (4-7)	5 (3-7)
Self-reported new knee injury at 5-11 y	2 (14)	1 (10)	1 (3)	1 (2)
Passive knee laxity	(n = 13)*^*b*^*	(n = 9)	(n = 27)	(n = 49)
Normal/nearly normal pivot-shift test finding	11 (85)	9 (100)	27 (100)	49 (100)
Normal/nearly normal Lachman test finding	9 (64)	5 (56)	27 (100)	48 (98)
ROA	(n = 14)	(n = 9)	(n = 26)	(n = 49)
Tibiofemoral	2 (14)	2 (22)	6 (23)	9 (18)
Patellofemoral	3 (21)	1 (11)	9 (35)	20 (41)
Sum of radiographic grades				
Mean ± SD	2 ± 2	2 ± 2	2 ± 2	3 ± 3
Median (IQR)	1 (0-2)	1 (1-3)	2 (1-4)	2 (1-3)

a Data are shown as n (%) unless otherwise indicated. ACL, anterior cruciate ligament; ACLR, anterior cruciate ligament reconstruction; IQR, interquartile range; KOOS, Knee Injury and Osteoarthritis Outcome Score; ROA, radiographic osteoarthritis; SF-36, 36-Item Short Form Health Survey.

b One participant had only results from the Lachman test.

**Figure 3.**
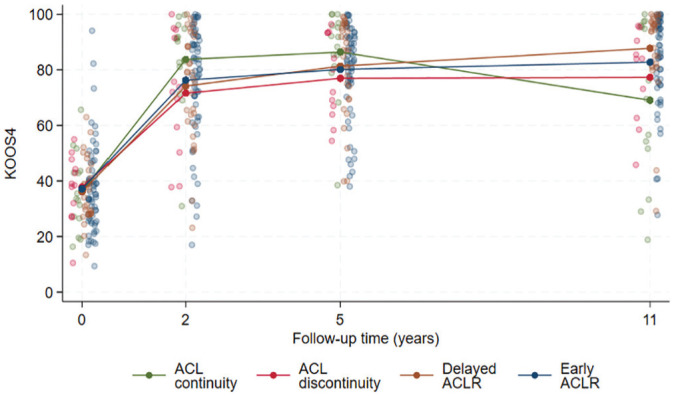
Individual and group mean values of Knee Injury and Osteoarthritis Outcome Score (KOOS_4_) scores at baseline and 2, 5, and 11 years’ follow-up by 5-year anterior cruciate ligament (ACL) continuity and treatment status. ACLR, anterior cruciate ligament reconstruction.

Adjusted analyses suggest that patients with ACL continuity on 5-year MRI reported worse 11-year KOOS_4_ scores compared with the delayed ACLR (adjusted mean difference, –20.2 [95% CI, –31.9 to −8.6]) and early ACLR (adjusted mean difference, –15.5 [95% CI, –26.4 to −4.7]) groups as well as similar or worse KOOS_4_ scores compared with patients with ACL discontinuity (adjusted mean difference, –11.4 [95% CI, –26.5 to 3.6]) (Table 3 and Figure 3). The sensitivity analysis using a censored regression (tobit) model resulted in similar findings to the primary analysis (Table 3). The Appendix (available in the online version of this article) presents the change in group mean values of the Tegner Activity Scale, SF-36 mental component summary, and SF-36 physical component summary over time (baseline and 2, 5, and 11 years’ follow-up).

**Table 3:** Adjusted Mean Differences in 11-Year KOOS_4_ Scores*^*a*^*

	ACL Continuity vs ACL Discontinuity	ACL Continuity vs Delayed ACLR	ACL Continuity vs Early ACLR
Linear regression	−11.4 (–26.5 to 3.6)	−20.2 (–31.9 to −8.6)	−15.5 (–26.4 to −4.7)
Tobit	−10.8 (–26.5 to 5.0)	−21.2 (–33.5 to −8.9)	−16.2 (–33.5 to −4.8)

a Data are shown as adjusted mean difference (95% CI). ACL, anterior cruciate ligament; ACLR, anterior cruciate ligament reconstruction; KOOS, Knee Injury and Osteoarthritis Outcome Score.

The proportion of participants with tibiofemoral ROA ranged from 14% (ACL continuity group) to 23% (delayed ACLR group), and the proportion of participants with patellofemoral ROA ranged from 11% (ACL discontinuity group) to 41% (early ACLR group) (Table 2). Patients who underwent exercise therapy first, irrespective of ACL continuity status or delayed ACLR, had a mean sum of radiographic grades of osteoarthritis at 11 years of 2 compared with 3 in the early ACLR group (Table 2).

## Discussion

Surprisingly, patients with ACL continuity at 5 years experienced comparable or worse 11-year outcomes compared with patients with ACL discontinuity and worse 11-year outcomes than those managed with early or delayed ACLR. This is despite the fact that patients with ACL continuity on 5-year MRI reported similar or better outcomes at 2- and 5-year follow-up compared with the other groups. These findings highlight the need for further large studies to understand the relationship between signs of ACL healing on MRI after an ACL rupture and long-term outcomes.

A recent study found that KANON trial participants with ACL continuity on 2-year MRI reported better 2-year outcomes (KOOS sports and recreation and KOOS quality of life subscale scores) than those with ACL discontinuity at 2 years as well as better outcomes than those in the early and delayed ACLR groups.^6^ Unlike the current study, at 2-year follow-up, ACL status on MRI was assessed at the same time that KOOS scores were obtained.^6^ Notably, we did not have MRI data at 11-year follow-up; thus, we used 5-year MRI findings and looked at the relationship with 11-year outcomes. It is possible that several participants with ACL continuity at 5 years no longer had ACL continuity at 11 years. Although we collected self-reported new knee injuries between 5 and 11 years and describe those that resulted in a consultation with a health care professional, relying on self-reports over a 6-year recall period is likely an unreliable method for capturing all relevant instability episodes and new knee trauma that could disrupt ACL continuity. Using self-reports, 2 participants with ACL continuity at 5 years recounted a new knee injury that caused them to consult a health care professional compared with 1 participant with ACL discontinuity at 5 years, 1 participant who underwent delayed ACLR, and 1 participant who underwent early ACLR. It is also possible that patients with ACL continuity with a history of ACL ruptures undergo more gradual changes in structure over time, which may be associated with reductions in knee function. An investigation of this possibility is outside the scope of the current study.

Patients with ACL continuity attended fewer rehabilitation visits for their ACL injury (mean, 32 ± 9 visits) compared with those with ACL discontinuity at 5 years (mean, 48 ± 34 visits) or those who underwent delayed or early ACLR (mean, 67 ± 37 visits). Considering that the progression of rehabilitation was goal based, the lower number of rehabilitation visits for those with ACL continuity could reflect better knee function and quality of life within the first 12 months of the injury, which could be attributed to regaining continuity of the ACL. At 2- and 5-year follow-up, patients with ACL continuity at 2 years were participating at a comparable or higher level of sports than those with ACL discontinuity or those who underwent ACLR.^6^ It is possible that regaining ACL continuity within 2 years of an ACL rupture resulted in improved knee function and greater knee confidence (reflected by better KOOS subscale scores^6^), leading to more exposure to high-risk sports and greater demands on the knee. Participation in cutting and pivoting sports after ACLR has been associated with an increased risk of graft ruptures and further knee injuries.^4^ It is plausible that the improved knee function and better quality of life in those with ACL continuity at 2 years led to more exposure to higher risk sports and more subsequent knee injuries. Activity levels at 11 years were similar or lower in the ACL continuity group compared with the other groups, which might reflect a reduction in activity participation between 5 and 11 years. Patients with ACL continuity reported a mean Tegner Activity Scale score of 6 at 2 years,^6^ a mean score of 5 at 5 years,^6^ and a mean score of 3 at 11 years. A similar decrease was seen in the other groups as well. Larger longitudinal cohorts are needed to explore the relationship between signs of ACL healing on MRI, patient-reported outcome scores, sports participation, and reinjury rates.

An important consideration is that not all ACLs that regain continuity after an ACL rupture, as observed on MRI, resemble a healthy, uninjured ACL.^5,6,9^ Because of the small number of participants in this group, we considered ACL continuity to include both a thick and taut ACL on MRI (resembling a healthy appearance) as well as a continuous but thin and/or elongated ACL. Findings from a study that investigated the potential for a knee brace to facilitate healing after ACL ruptures suggest that patients with a thick and taut continuous ACL report better outcomes and have reduced knee laxity compared with patients with a thin and/or elongated continuous ACL.^5^ It is possible that a thinned and/or elongated ACL places the knee at a greater risk of further knee injuries or reruptures, which may lead to worse knee function, symptoms, and quality of life in the long term. However, even a thinned and/or elongated ACL with a continuous appearance on MRI should theoretically not lead to worse knee function than a discontinuous ACL. Furthermore, participants in the KANON trial were subject to repetitive assessments of mechanical knee laxity within the first 12 months of the injury, including Lachman and pivot-shift tests. The effect of such testing on ACL tissue during the early phases of rehabilitation is unknown.

### Strengths and Limitations

Within the KANON trial, no participants or treating clinicians were informed of the ACL continuity status. This is relevant because receiving news that their ACL has “healed” could affect patients’ perception of their knee as well as their knee confidence and willingness to participate in high-risk sports. Another strength of this study is the long-term follow-up. To our knowledge, this is the first study to investigate signs of ACL continuity on MRI in relation to long-term outcomes beyond 5 years. A key limitation is the small sample size in the ACL continuity and ACL discontinuity groups, which leads to imprecise estimates. Although an 87% retention rate at 11 years exceeds the typical long-term follow-up in trials, the loss of participants between 5- and 11-year follow-up could introduce selection bias to the remaining trial population and produce biased estimates.^2^ An acute ACL rupture was confirmed by MRI for all but 1 participant (who had a positive pivot-shift test finding) using the methods of MRI that were common practice at the time. For the purposes of this secondary analysis, we reanalyzed all baseline MRI scans using the more advanced ACLOAS criteria. This resulted in 2 participants being reclassified as having a high-grade partial ACL rupture (ACLOAS grade 1) at baseline. Both participants had been randomized to undergo initial exercise therapy and optional delayed ACLR and had positive pivot-shift test findings at baseline. The lack of MRI data at 11 years is also a limitation, as is the reliance on self-reports of new knee injuries between 5 and 11 years. We also did not collect measurements of passive knee laxity at 11-year follow-up. Further research is needed to understand the relationship between ACL structure on MRI and passive and functional stability of the knee.

## Conclusion

We found that participants with ACL continuity on MRI at 5 years after an acute ACL rupture reported similar or worse patient-reported outcomes at 11 years after the injury compared with participants with ACL discontinuity and those managed with early or delayed ACLR. In contrast, patients with signs of ACL continuity had numerically similar or fewer signs of ROA at 11 years compared with those who were managed surgically. The small sample size and lack of MRI data from 11-year follow-up contribute to the uncertainty around these long-term outcomes and their generalizability.

## Supplemental Material

sj-pdf-1-ajs-10.1177_03635465251339061 – Supplemental material for Association Between ACL Continuity on Magnetic Resonance Imaging at 5 Years After an Acute ACL Rupture and 11-Year Outcomes: A Secondary Analysis From the KANON Trial Supplemental material, sj-pdf-1-ajs-10.1177_03635465251339061 for Association Between ACL Continuity on Magnetic Resonance Imaging at 5 Years After an Acute ACL Rupture and 11-Year Outcomes: A Secondary Analysis From the KANON Trial by Stephanie R. Filbay, Frank Roemer, Ewa M. Roos, Aleksandra Turkiewicz, Richard Frobell, L. Stefan Lohmander and Martin Englund in The American Journal of Sports Medicine
