# Supplementary material for: Association Between ACL Continuity on Magnetic Resonance Imaging at 5 Years After an Acute ACL Rupture and 11-Year Outcomes: A Secondary Analysis From the KANON Trial
Source: Am J Sports Med. 2025 May 19;53(8):1893–900. doi: 10.1177/03635465251339061 (PMC12185899; doi:10.1177/03635465251339061)
Supplement: sj-pdf-1-ajs-10.1177_03635465251339061 – Supplemental material for Association Between ACL Continuity on Magnetic Resonance Imaging at 5 Years After an Acute ACL Rupture and 11-Year Outcomes: A Secondary Analysis From the KANON Trial [file sj-pdf-1-ajs-10.1177_03635465251339061.pdf]

IS ANTERIOR CRUCIATE LIGAMENT (ACL) CONTINUITY ON MRI 5 YEARS AFTER ACUTE ACL RUPTURE ASSOCIATED WITH 11-YEAR OUTCOMES? A SECONDARY ANALYSIS FROM THE KANON TRIAL

Appendix

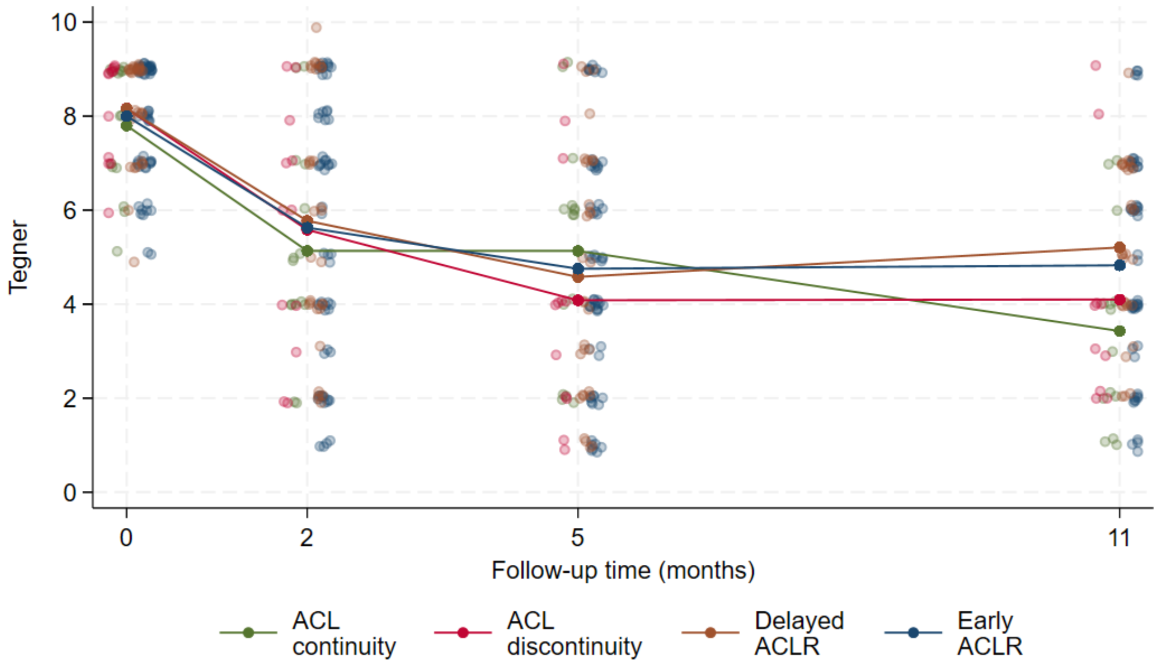

Figure A1. Individual values and group means of Tegner Activity Scale scores at baseline, 2, 5 and 11 years of follow-up, by 5-year ACL continuity and treatment status

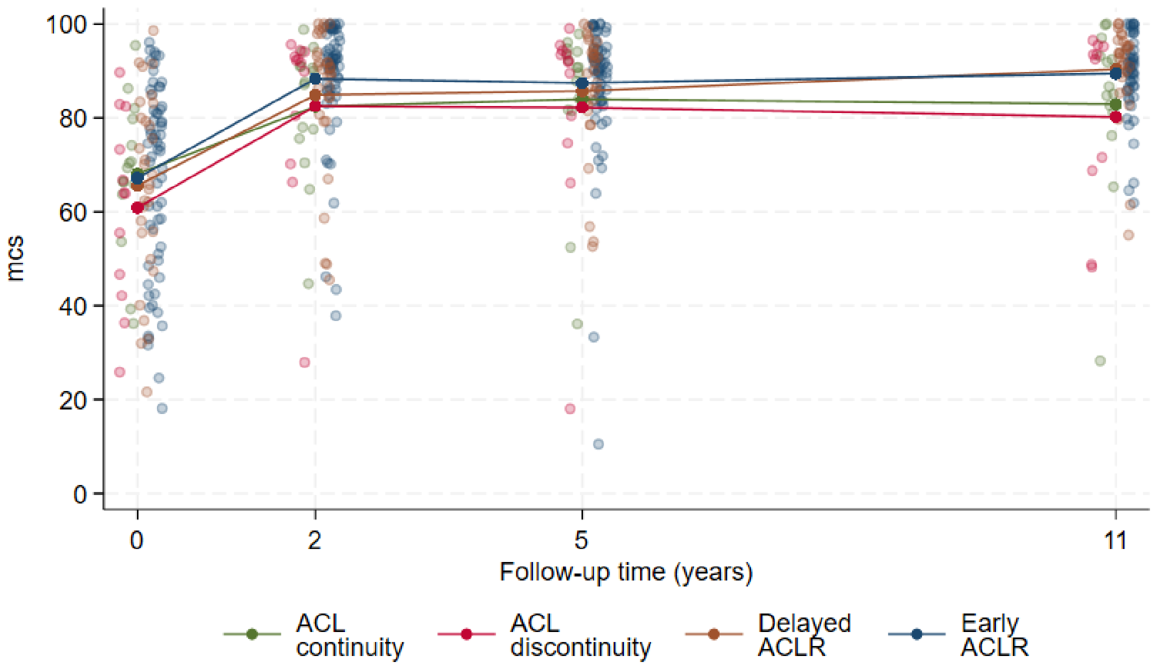

Figure A2. Individual values and group means of SF36 MCS scores at baseline, 2, 5 and 11 years of follow-up, by 5-year ACL continuity and treatment status

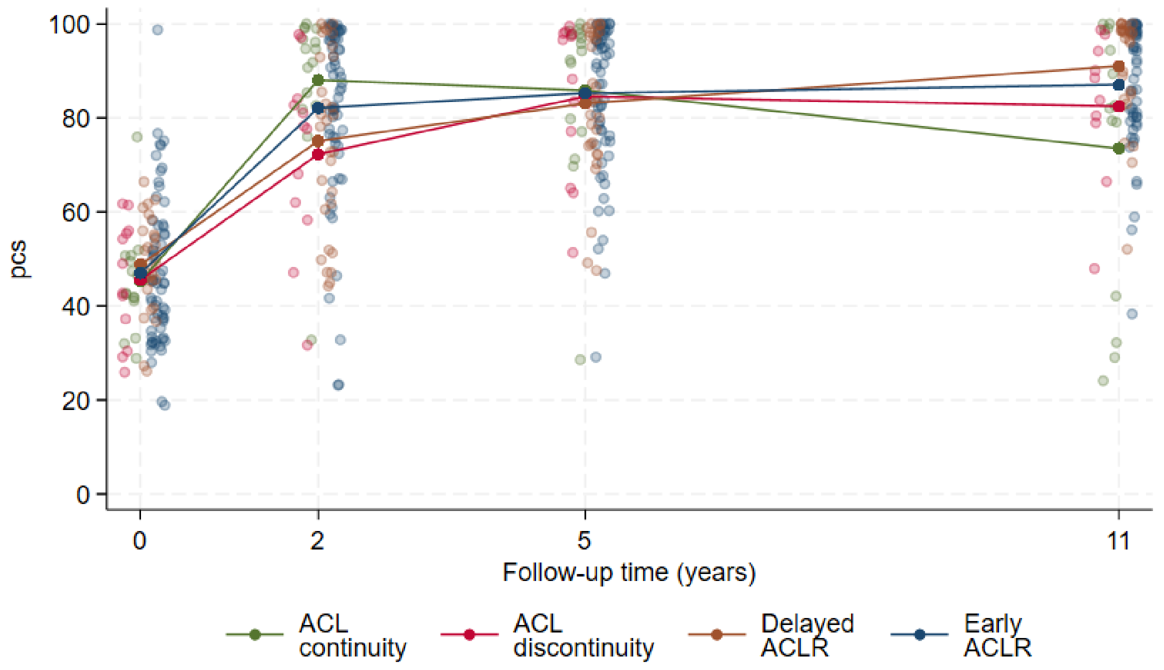

Figure A3. Individual values and group means of SF36 PCS scores at baseline, 2, 5 and 11 years of follow-up, by 5-year ACL continuity and treatment status
